# A novel method of inducing thrombosis in target arteries using coronary stents for therapeutic occlusion

**DOI:** 10.1186/s13104-018-3511-6

**Published:** 2018-07-03

**Authors:** Mark Christopher Arokiaraj

**Affiliations:** Cardiology, Pondicherry Institute of Medical Sciences, Kalapet, India

**Keywords:** Peripheral arterial embolization, Coronary stents, Coils

## Abstract

**Objectives:**

Peripheral artery embolizations are often required for therapeutic purposes. The therapeutic embolizations are usually performed using embolization particles and coils. These procedures are also associated with complications. Coils and their delivery cables, and the expertise are not always available in all catheterization centers. Hence, a novel, simple controlled technique for arterial closure would be useful in emergency settings.

**Results:**

Following is a report of seven cases where embolization was performed successfully on an emergency basis after stenting using coronary stents for better closure of the target artery. Six patients underwent bronchial artery embolization for recurrent massive hemoptysis, and one another patient had pseudoaneurysm following percutaneous nephrolithotomy, and presented with hematuria and persistent and increasing blood discharge in drainage catheter. The stents were deployed in the target artery using 6F Judkin’s diagnostic catheter over the 014 wires, which are easily available in all cardiac catheterization laboratories. This is a novel method to establish a metal platform inside the target arteries using coronary stents for better closure of the target arteries in combination with embolization techniques. The procedures were performed as lifesaving measures when small coils and the delivery cables were not available at the time of the procedures.

**Electronic supplementary material:**

The online version of this article (10.1186/s13104-018-3511-6) contains supplementary material, which is available to authorized users.

## Introduction

Peripheral arterial embolizations are often required in routine clinical practice [1], and frequently these procedures are life-saving. Embolization with coils, glue, gel foam, polyvinyl alcohol (PVA) particles and vascular plugs are used frequently for these purposes [1]. Coil deployment to the target artery is the procedure of choice [2]. However, often the expertise and personnel for coil deployment, and the appropriate delivery cables are not frequently available in all centers. Hence, a novel, simple method available in all the centers, which could be used by any interventionist, would be frequently helpful as a life saving measure.

## Main text

Following is a report of seven cases who underwent stenting and induction of thrombosis in the arteries for the closure of the artery for therapeutic purposes.

### Patient 1

**Fig. 1:**
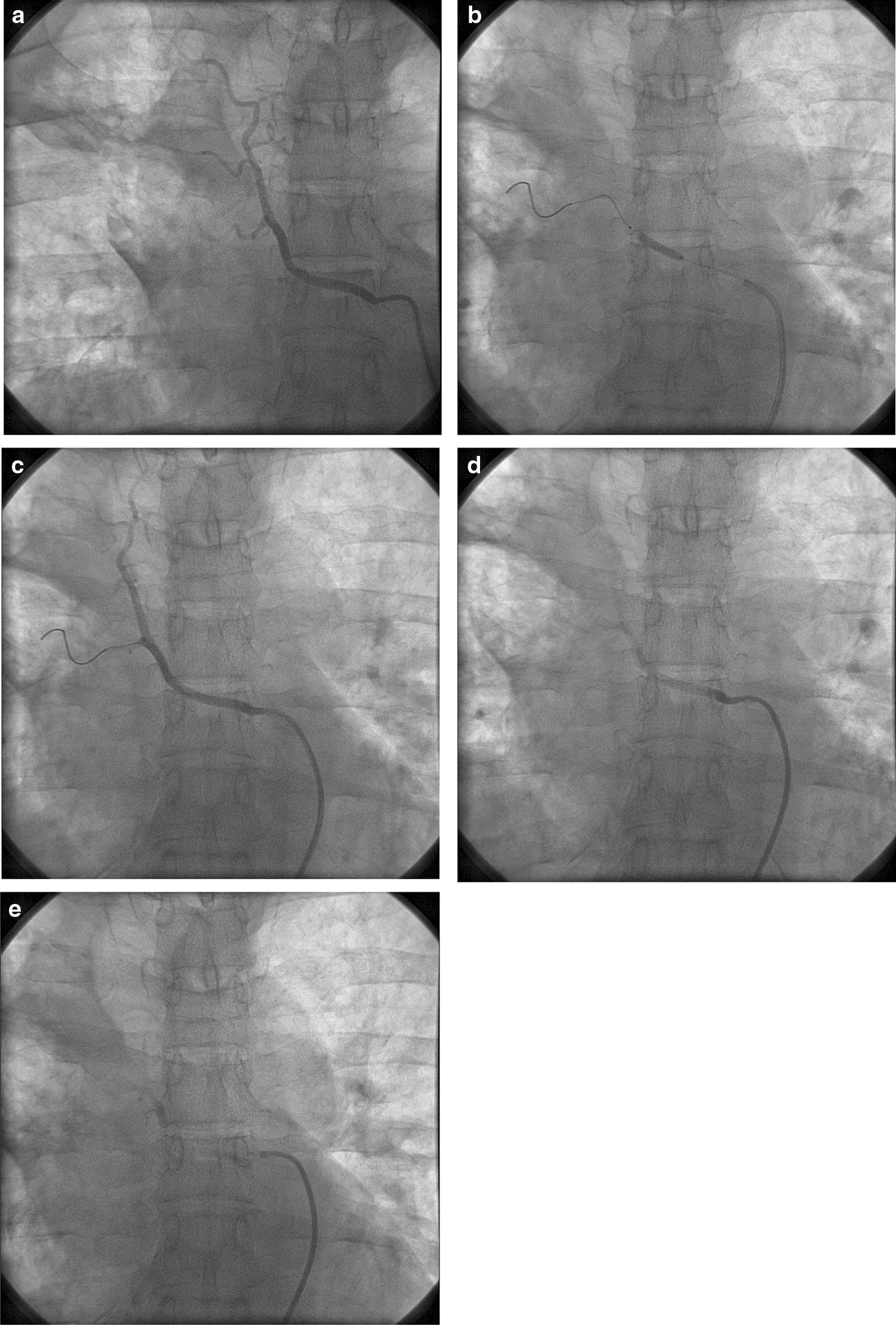
Bronchial artery embolization performed after stenting (**b**) of the enlarged bronchial artery showing complete occlusion (**e**). **a** The large bronchial artery, **b** stenting of the artery, **c** injection of embolization materials, **d** near total occlusion and **e** complete occlusion of the artery

The bronchial artery was identified as a feeder in a patient who presented with massive hemoptysis. The 45 year old male had multiple bouts of large volume hemoptysis, and therefore, bronchial artery embolization was planned. The bronchial artery was initially embolized with gel foam and PVA (polyvinyl alcohol 200 μm, Cook Medical) particles using a Cordis right Judkin’s 6F diagnostic catheter and iodixanol as contrast. As the artery was large coil embolization was preferred (Additional file 1: Patient 1a). For better occlusion as the arterial caliber was large a coronary stent (Ultimaster 2.5 mm × 18 mm) was deployed in the feeding bronchial artery (Additional file 2: Patient 1b), and prolonged balloon inflation using the same stent balloon at low pressure (7 atm). After that embolization was performed with PVA particles and gel foam combination (Additional file 3: Patient 1c). Subsequently, the feeding artery was closed (Fig. 1d and e, Additional file 4). The patient had severe chest pain immediately after the procedure, and the pain was controlled with opioid injections. The patient subsequently recovered, and on follow-up, for 11 m there was no hemoptysis.

### Patient 2

A 40 year lady, who was a known case of pulmonary tuberculosis presented with recurrent episodes of massive hemoptysis. Her chest X-ray showed infiltrates in the right upper lobe. Bronchial artery embolization was planned, and a bronchial angiogram was performed. The angiogram showed a large bronchial artery with collateral supply in the right upper lobe area (Additional file 5: Patient 2a). The feeding arterial occlusion was planned. The bronchial artery was engaged with right 6F Judkin’s diagnostic catheter, and the embolization was initially performed with gel foam and PVA particles (300 μm, Additional file 6: Patient 2b). An O14 wire was inserted, and a stent (Yukon Choice, 2.5 mm × 8 mm) was deployed in the mid-segment of the bronchial artery at low pressure (7 atm, Additional file 7: 2c). After that, another stent (Endeavor Sprint, 2.5 mm × 14 mm) was deployed in the proximal segment of the artery and prolonged inflation was given at 6 atm using the stent balloon (Additional file 8: Patient 2d). After that, the stent balloon was removed, and PVA particles and gel foam were injected to ensure complete occlusion of the artery (Additional file 9: Patient 2e). In this case, since the vessel was tortuous two short stents were used as a single long stent may not be trackable easily. The patient had severe chest discomfort which reduced 1 h after the procedure. Also, to alleviate her chest pain she required a small dose of fentanyl. Hemoptysis subsequently subsided, and the patient’s condition improved. This patient is currently under follow for 6 m without symptoms.

### Patient 3

**Fig. 2:**
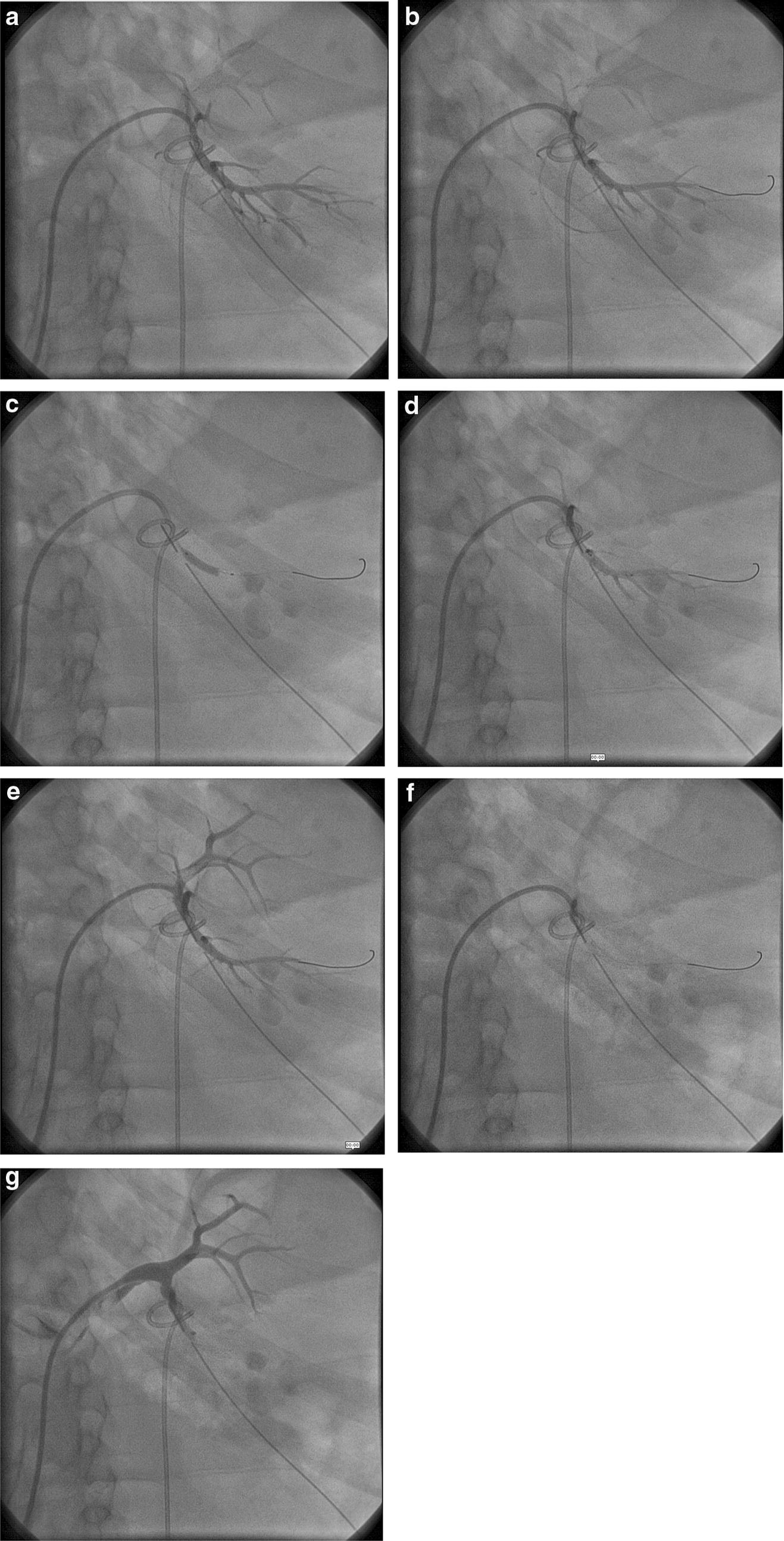
Segmental renal arterial embolization after stenting closing the pseudoaneurysm in its mid-segment. Panel **a** shows the aneurysm, panel **b** shows placement of stent across pseudoaneurysm, panel **c** shows stent deployment, and panels **d** and **e** show reduction in pseudoaneurysm. Panels **f** and **g** show complete occlusion subsequently after embolisation

The 50 year male patient underwent percutaneous nephrolithotomy due to obstructive nephropathy induced by a renal calculus. The patient had a percutaneous drainage catheter, which showed persistent blood discharge in the drainage catheter, which was progressively increasing. This patient also had significant hematuria. The renal angiogram was performed on the patient using 6F Judkin’s right diagnostic catheter, and the patient had pulsatile bleeding/pseudoaneurysm in the lower segmental artery as noticed by extravasation of contrast from the lower segmental artery (Additional file 10: Patient 3a). Hence, in this case, a coronary stent was deployed in the bleeding artery across the pseudoaneurysm using a 014 wire and Nobori 3.0 mm × 15 mm and Cordis 6F right Judkin’s diagnostic catheter (Fig. 2c, d, Additional file 11: Patient 3b, Additional file 12: Patient 3c). Prolonged low-pressure stent balloon inflation was performed for 8 min at 7 atm pressure using the same stent balloon. After that, the balloon was removed, and therapeutic embolization was performed using gel foam and PVA particles (300 μm, Cook Medical) in the segmental artery using the JR 6F diagnostic catheter and keeping the 014 wire in the segmental artery (Fig. 2e, f, Additional file 13: Patient 3d, Additional file 14: Patient e). Angiogram revealed complete occlusion of the lower polar artery (Fig. 2g, Additional file 15: Patient 3f). The patient had severe loin pain immediately after the procedure, which was managed with fentanyl injection. Subsequently, the discharge from the percutaneous drainage was reduced, and the drainage tube was removed after 3 days. This patient is being followed up for 1 year, and there are no bleeding manifestations.

### Patient 4

Patient 4 who was a 45 year male presented with aspergillosis of lung and patient had recurrent episodes of large volume hemoptysis. He was admitted with another bout of massive hemoptysis. The patient underwenÚt bronchial arterial embolization 5 years before, and a 035′ wire cut segment was placed at the proximal end of the bronchial artery in the past. Since the patient presented with recurrent hemoptysis the bronchial arterial embolization was performed with the similar technique with PVA particles and gel foam and a coronary stent was placed in the proximal segment of the artery (Additional file 16: Patient 4a, Additional file 17: Patient 4b, Additional file 18: Patient 4c, Additional file 19: Patient 4d). The patient is followed up for 3 months, and there were no other episodes of hemoptysis. For definitive therapy of aspergillosis, the patient was not willing for surgery. Hence, he was discharged with a course of oral itraconazole.

### Patient 5

Patient 5 was a 50 year old male, had pulmonary tuberculosis in the past with destroyed left lung, and this patient also had a bronchial artery embolization in the past with gel foam only, which was performed 4 years before. The patient was admitted with massive hemoptysis. A large bronchial feeder was identified in the left upper lobe, and embolization was performed with PVA particles and followed by gel foam. At the end of the procedure a stent was placed (endeavor 2.5 mm × 18 mm) at 8 atm, and final closure was performed with PVA particles followed by gel foam with good results (Additional file 20: Patient 5a Additional file 21: Patient 5b, Additional file 22: Patient 5c, Additional file 23: Patient 5d, Additional file 24: Patient 5e). The patient had severe chest pain and mild hemoptysis at the end of the procedure. The patient is being followed up for 3 months, and there are no episodes of hemoptysis.

### Patient 6

Patient 6 was a 48 year male, and he had recurrent episodes of hemoptysis every year for the past 10 years. During this episode, patient had massive hemoptysis of about 300–400 ml associated with pre-syncope. He was evaluated by CT chest with contrast and bronchoscopy, which did not reveal any source of hemoptysis. The bronchial angiogram revealed extensive broncho-pulmonary collaterals with a large bronchial artery (3.5 mm). Embolization was performed with PVA particles, and a small stent (Yukon Choice 2.25 mm × 18 mm) was placed in the proximal segment of the artery through a Cordis 6F left Judkin's diagnotic catheter. The stent was deployed at 6 atm. Subsequently, gel foam and PVA particles were injected, and the vessel was closed (Additional file 25: Patient 6a, Additional file 26: Patient 6b, Additional file 27: Patient 6c, Additional file 28: Patient 6d). The patient is under follow up for 2 months without symptoms.

### Patient 7

Patient 7 was a 60 year lady who had recurrent episodes of hemoptysis, and CT chest showed bronchiectasis of right middle and lower lobes. The patient presented with massive hemoptysis and patient was taken up for an urgent bronchial angiogram. The bronchial angiogram showed a large bronchial artery with distal collaterals. Embolization was performed with the similar technique, and a stent was placed (Yukon PC 2.75 mm × 28 mm) at low-pressure inflation of 6 atm., and complete closure of the vessel was achieved (Additional file 29: Patient 7a, Additional file 30: Patient 7b, Additional file 31: Patient 7c, Additional file 32: Patient 7d).

In all the cases the procedures were performed within 24 h of admission to the hospital, and all patients had significant life-threatening bleeding episodes. Unfractionated heparin of about 500 to 1000 U in total was used during the procedures. All procedures were performed through femoral route, and the sheath was removed immediately after the procedure. None of the patients developed peripheral vascular or spinal complications after the procedures. Also, none of the patients had episodes of hemoptysis or bleeding on follow-up.

## Discussion

Bronchial artery embolization (BAE) was first described by Rémy et al. in 1973 [3], and it is now considered a first-line treatment for most cases of massive hemoptysis [4, 5]. The technique performed in the above seven cases is a new technique of embolization after deployment of a stent in the bleeding artery. This is more effective than embolization with only particles as the stent could provide a skeleton for a stronger occlusion with a metal platform.

### The stent as an embolization platform

Coils are available, and they can be used effectively for the closure of the arteries. However, coil technique requires deployment device, coils and also technical expertise in coil deployments. These may not be available in all centers especially in the peripheral catheterization centers. Also, the controlled-release detachable coils are not available or marketed in all countries. Moreover, the stent deployments can be very selective to arterial branches and controlled than coil usage. The deployment of a stent is simple and fast, and a routine diagnostic 6F right Judkin’s catheter is available in most centers and easy to use. Also, frequently these procedures are performed as a lifesaving method, for example, to prevent a massive uterine arterial bleeding or massive hemoptysis. Any coronary/peripheral interventionist in an emergency setting with limited hardware could use these stents. These stents can be easily taken through JR 6F diagnostic catheters over a 014 wire to any selected arteries for therapeutic purposes. The manipulation of JR diagnostic catheters over 014 wire makes it more selective for injection of embolization particles than without the wire, as these catheters can be throttled inside the target artery across the 014 wires (Figs. 1 and 2). Technically coils are relatively difficult to deploy in interiorly located arteries than coronary stents, which are more trackable to the target vessel than coils. Stents also tend to reduce the quantum of embolization materials, which need to be used with caution as spillage during injection leads to thrombotic complications in the neighboring and the distal structures. Also, micro-catheters are not required using this technique. Bare metal stents with thicker struts are a better choice than drug-eluting stents for this purpose though bare metal stents were not available in our center when these procedures were performed. A thicker stent, which is undersized to the artery and low-pressure deployment leading to under-expansion and usage of multiple stents are preferable for this purpose. These would ensure a ‘coil effect’ or tissue reaction essential for restenosis and promote clot formation in the target vessel [6, 7].

All seven patients had significant pain immediately after the injection and occlusion of the artery, which indicates that the occlusion is complete and the pain experienced was more than in patients with the injection of the embolization particles alone. This also indicates the better efficacy of the procedures in combination with stents and embolization materials than with the materials alone.

Renal artery embolization is commonly performed with embolization glues, for example, *N*-butyl cyanoacrylate [8, 9]. This is the first report of using coronary stents for selective embolization of the bleeding arteries. This technique could be used for other peripheral arterial embolization also, for example, uterine artery embolization. This is very useful when coil closure method is not available or not feasible due to lack of technical expertise, which is not infrequent.

### Safety profile

There is no previous report of using coronary stents for this purpose of therapeutic closure of arteries. The potential and significant side-effect of any bronchial artery embolization therapy is paraparesis or neurological events [4, 10], which was not seen in any of the patients in this case series with this technique of stent deployment and embolization.

## Limitations

The number of patients treated by this technique is small, and in this series, in total, only eight stents were used. Further studies need to be performed in large numbers of patients, with long-term follow-up and the results need to be validated with statistical analysis. Nevertheless, as a technique it is simple, and any interventionist in any emergency setting could perform these procedures with ease and could be life-saving.

## Additional files

The additional video file of the patients showing the initial bronchial angiogram/feeder using 5F radial Tiger catheter and initial embolization was performed. Subsequently using 6F Cordis Judkins right diagnostic catheter over a 014 wire, coronary stents were deployed at low pressure and final embolization was performed in the artery. Patient 3 underwent stenting and embolisation to the left lower polar renal artery.

**Additional file 1.** Patient 1a.

**Additional file 2.** Patient 1b.

**Additional file 3.** Patient 1c.

**Additional file 4.** Patient 1d.

**Additional file 5.** Patient 2a.

**Additional file 6.** Patient 2b.

**Additional file 7.** Patient 2c.

**Additional file 8.** Patient 2d.

**Additional file 9.** Patient 2e.

**Additional file 10.** Patient 3a.

**Additional file 11.** Patient 3b.

**Additional file 12.** Patient 3c.

**Additional file 13.** Patient 3d.

**Additional file 14.** Patient 3e.

**Additional file 15.** Patient 3f.

**Additional file 16.** Patient 4a.

**Additional file 17.** Patient 4b.

**Additional file 18.** Patient 4c.

**Additional file 19.** Patient 4d.

**Additional file 20.** Patient 5a.

**Additional file 21.** Patient 5b.

**Additional file 22.** Patient 5c.

**Additional file 23.** Patient 5d.

**Additional file 24.** Patient 5e.

**Additional file 25.** Patient 6a.

**Additional file 26.** Patient 6b.

**Additional file 27.** Patient 6c.

**Additional file 28.** Patient 6d.

**Additional file 29.** Patient 7a.

**Additional file 30.** Patient 7b.

**Additional file 31.** Patient 7c.

**Additional file 32.** Patient 7d.

## Abbreviations

BAE: bronchial artery embolization
PVA: polyvinyl alcohol
